# Gene Expression Profiling Confirms the Dosage-Dependent Additive Neuroprotective Effects of Jasminoidin in a Mouse Model of Ischemia-Reperfusion Injury

**DOI:** 10.1155/2018/2785636

**Published:** 2018-05-16

**Authors:** Haixia Li, Jingtao Wang, Pengqian Wang, Yingying Zhang, Jun Liu, Yanan Yu, Bing Li, Zhong Wang

**Affiliations:** ^1^Guang'anmen Hospital, China Academy of Chinese Medical Sciences, Beijing 100700, China; ^2^Beijing University of Chinese Medicine, Beijing 100029, China; ^3^Institute of Basic Research in Clinical Medicine, China Academy of Chinese Medical Sciences, Beijing 100700, China; ^4^Dongzhimen Hospital, Beijing University of Chinese Medicine, Beijing 100700, China; ^5^Institute of Information on Traditional Chinese Medicine, China Academy of Chinese Medical Sciences, Beijing 100700, China

## Abstract

Recent evidence demonstrates that a double dose of Jasminoidin (2·JA) is more effective than Jasminoidin (JA) in cerebral ischemia therapy, but its dosage-effect mechanisms are unclear. In this study, the software GeneGo MetaCore was used to perform pathway analysis of the differentially expressed genes obtained in microarrays of mice belonging to four groups (Sham, Vehicle, JA, and 2·JA), aiming to elucidate differences in JA and 2·JA's dose-dependent pharmacological mechanism from a system's perspective. The top 10 enriched pathways in the 2·JA condition were mainly involved in neuroprotection (70% of the pathways), apoptosis and survival (40%), and anti-inflammation (20%), while JA induced pathways were mainly involved in apoptosis and survival (60%), anti-inflammation (20%), and lipid metabolism (20%). Regarding shared pathways and processes, 3, 1, and 3 pathways overlapped between the Vehicle and JA, Vehicle and 2·JA, and JA and 2·JA conditions, respectively; for the top ten overlapped processes these numbers were 3, 0, and 4, respectively. The common pathways and processes in the 2·JA condition included differentially expressed genes significantly different from those in JA. Seven representative pathways were only activated by 2·JA, such as* Gamma-Secretase regulation of neuronal cell development. *Process network comparison indicated that significant nodes, such as* alpha-MSH*,* ACTH*,* PKR1*, and* WNT*, were involved in the pharmacological mechanism of 2·JA. Function distribution was different between JA and 2·JA groups, indicating a dosage additive mechanism in cerebral ischemia treatment. Such systemic approach based on whole-genome multiple pathways and networks may provide an effective and alternative approach to identify alterations underlining dosage-dependent therapeutic benefits of pharmacological compounds on complex disease processes.

## 1. Introduction

Cerebral ischemia is a fatal disease, in which there is insufficient blood flow to the brain to meet metabolic demand; therefore, it leads to cerebral hypoxia and subsequent brain tissue death. The biochemical and molecular mechanisms triggered by ischemia, including reperfusion injuries and alterations in multiple genes, can cumulatively lead to progressive neurological damage and cell death [1]. Jasminoidin (JA) is a herbal extract that has shown to have protective effects in cases of ischemia, in a dosage-dependent manner [2]. Previous studies in rats with cerebral ischemia have shown that JA's pharmacological mechanisms involve multiple pathways [3], but its dosage effect remains poorly understood. It is therefore essential to elucidate the relationship between the complex pharmacological mechanisms and different JA dosages in cerebral ischemia, that is, assessing whether and how altered dosages affect the protective drug mechanisms.

JA is commonly used in traditional Chinese and clinical medicine to treat stroke, especially in Qingkailing [4]. The pharmacological properties of JA include neuroprotective, anti-inflammatory, cholagogue, anticoagulant, and anti-oxidative effects [2, 5]. Previous studies have demonstrated that JA affects multiple signaling cascades such as ATP-binding cassette (ABC) transporter [6], NF-*κ*B [7], PI3K [8], TLR4 [9], and mitogen-activated protein kinase (MAPK) [6] signaling pathways.

In order to assess the similarities and differences of pharmacological mechanisms elicited by different JA dosages, we used the GeneGo MetaCore™ software [10], which was based on well manually curated database of protein-protein, signaling, and others, to analyze the data obtained from mice brain expression microarrays in our previous study [2]. This high-throughput data supported software was used to identify the differential pathway networks affected by JA in a rodent model of cerebral ischemia-reperfusion injury. The aim of this study was to determine the JA's dosage effect on its pharmacological mechanism in the treatment of cerebral ischemia, through analysis of signaling pathways and networks activated after JA and 2·JA administration.

## 2. Results

### 2.1. Pharmacodynamic Results

**Figure 1:**
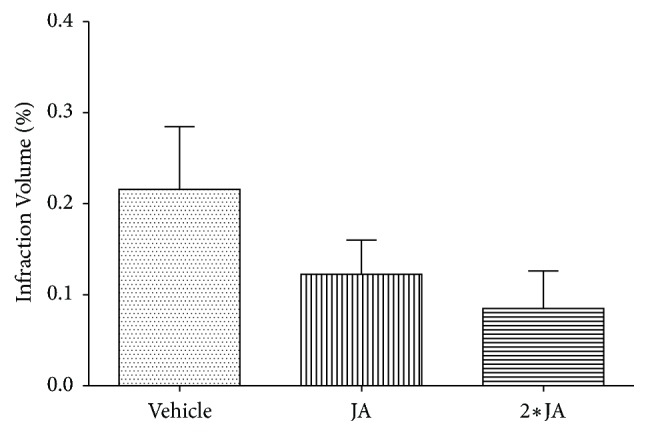
Variation of infarction volume among different groups. Both 2·JA and JA were effective in reducing the ischemic infarction volume.

In our previous study, histological analysis observed that the Nissl bodies in hippocampus samples of Vehicle were considerably depleted, while the loss of Nissl bodies in JA group was significantly alleviated [11]. As shown in Figure 1, both 2·JA and JA were effective in reducing ischemic infarction volume when compared with the Vehicle group, with 2·JA showing the lowest level and thus the best protective result.

### 2.2. Comparison of Enriched Pathways among Treatment and Vehicle Groups

**Figure 2:**
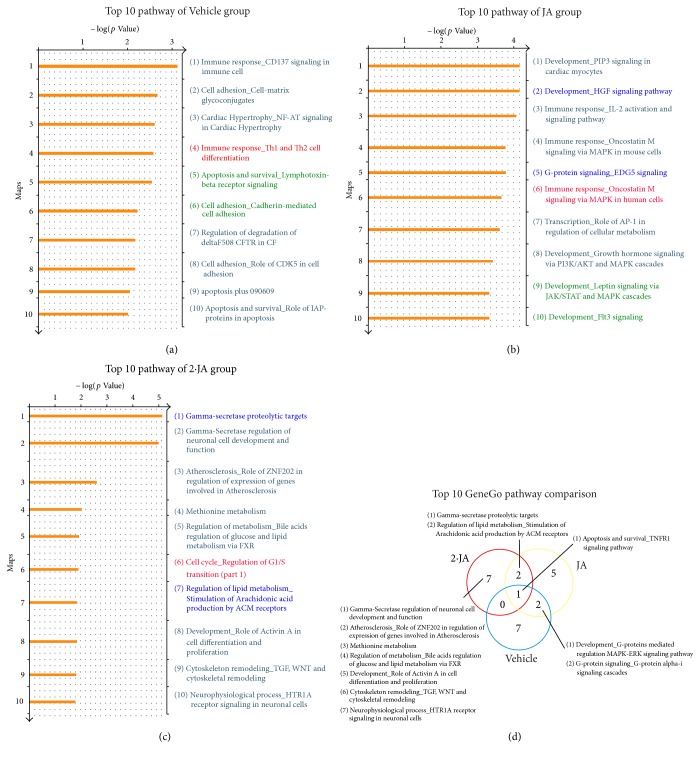
Pathway profiles in different groups analyzed with GeneGo. (a–c) The top 10 GeneGo pathways of each group are shown; the overlapping cascades between Vehicle and JA (2, in green) and JA and 2·JA (2, in blue) and among the three groups (1, in red) are marked; no overlapping pathways were shared between Vehicle and 2·JA groups. (d) Overlapping and specific pathways of each group are presented in a Venn diagram.

The pathways enriched in the Vehicle group were first examined to elucidate the pharmacological meaning of each treatment. Those statistically significant were identified by GeneGo MetaCore. The top 10 enriched pathways in the Vehicle, JA, and 2·JA groups are shown in Figures 2(a)–2(c), with each item ranked by calculated *p* values. As shown in the Venn diagram of Figure 2(d), 3 top pathways overlapped between the JA and Vehicle groups, while 1 was found in both 2·JA and Vehicle groups. Of note, the* G-protein signaling_G-protein alpha-i signaling cascades *pathway found in the Vehicle and JA groups was not in 2·JA's top ten pathways. Besides, the a*poptosis and survival_TNFR1 signaling pathway* was shared by all 3 groups (Figure 2(d)); importantly, JA and 2·JA treated animals shared only three top pathways, with the remaining seven exclusively enriched in the 2·JA group.

The pathways exclusively enriched in the JA and 2·JA groups might be involved in the pharmacological mechanisms of these regimens in the treatment of cerebral ischemia.

### 2.3. Comparison of Enriched Process Networks among Treatment and Vehicle Groups

**Figure 3:**
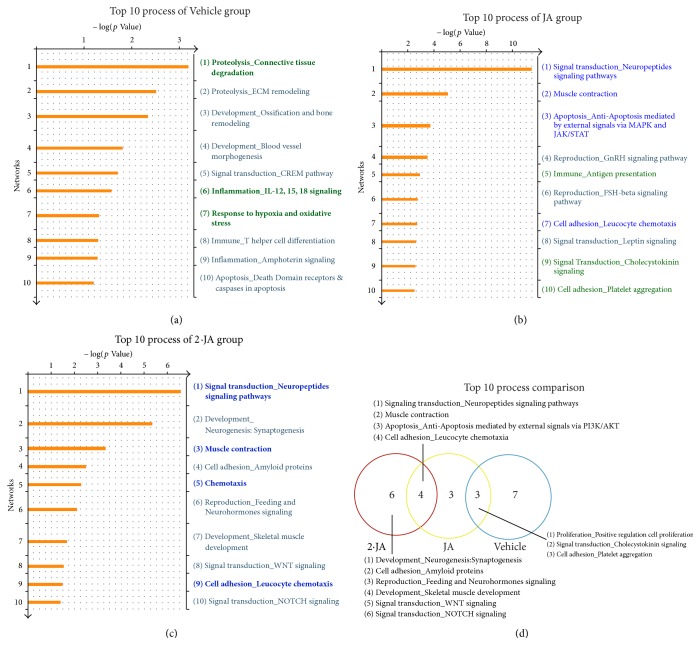
Process networks annotated by GeneGo for the Vehicle, JA, and 2·JA groups. (a–c) The top 10 GeneGo process networks of each group are shown; the overlapping cascades between Vehicle and JA (3, in green) and JA and 2·JA (4, in blue) are marked; no overlapping networks were shared between Vehicle and 2·JA. (d) Overlapping and specific process networks of each group are presented in a Venn diagram.

The process networks enriched in various groups were also identified by GeneGo MetaCore. The top 10 enriched process networks in the Vehicle, JA, and 2·JA groups are shown in Figures 3(a)–3(c), with each item ranked by calculated *p* values. Interestingly, 3 process networks overlapped between the JA and Vehicle groups (Figure 3(d)), including* signaling transduction_Cholecystokinin signaling*. Some of the processes found in the Vehicle group were changed by JA or 2·JA treatment through gene expression level changes. Importantly, no overlapping process network was found between the Vehicle and 2·JA groups. As for the pathways, the process networks exclusively enriched in the JA and 2·JA groups might be involved in the pharmacological mechanisms of respective regimens in the treatment of cerebral ischemia.

### 2.4. Comparison of JA and 2·JA Induced Changes

#### 2.4.1. Enriched Pathway Comparison between JA and 2·JA

The 3 overlapping pathways between the JA and 2·JA groups included* Gamma-Secretase proteolytic targets, Apoptosis and survival_TNFR1 signaling pathway*, and* regulation of lipid metabolism_Stimulation of arachidonic acid production by ACM receptors*. A total of 5 and 7 top 10 ranked pathways were exclusively represented in the JA and 2·JA groups, respectively. For instance,* G-protein signaling_EDG5 signaling* was found in the JA group list, while* Gamma-Secretase regulation of neuronal cell development and function, Development_Role of Activin A in cell differentiation and proliferation*, and* Cytoskeleton remodeling_TGF-β, WNT, and cytoskeletal remodeling* were represented in the 2·JA group (Figure 2(d)).

**Figure 4:**
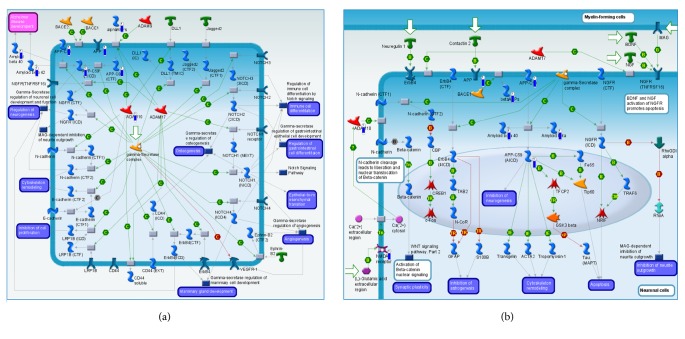
Two pathways of genes altered in the 2·JA group.* Gamma-Secretase proteolytic targets* (a) and* Gamma-Secretase regulation of neuronal cell development and function* (b). The map legend can be viewed at http://lsresearch.thomsonreuters.com/static/uploads/files/2014-05/MetaCoreQuickReferenceGuide.pdf.

Take two significant pathways in the 2·JA group as examples, that is,* Gamma-Secretase proteolytic targets* (Figure 4(a)) and* Gamma-Secretase regulation of neuronal cell development and function* (Figure 4(b)). In these two pathways, 5 out of 8 downregulated targets overlapped, including* ADAM10, APP, Amyloid beta 40, AICD*, and* alpha APPs*; other molecules such as CTFs were only found in the* Gamma-Secretase proteolytic targets pathway*, whereas beta APPs and NMDA receptor were only present in the other pathway.

#### 2.4.2. Comparison of Process Networks between JA and 2·JA Groups

Four process networks were overlapped between the top 10 processes of the JA and 2·JA groups, for example,* signal transduction-Neuropeptides signaling pathways* (Figure 3(d)). It is notable that 2·JA group had 6 exclusive and representative processes, such as the* Development_Neurogenesis: Synaptogenesis* and* cell adhesion_Amyloid proteins*. Besides, JA group displayed 3 exclusive processes.

**Figure 5:**
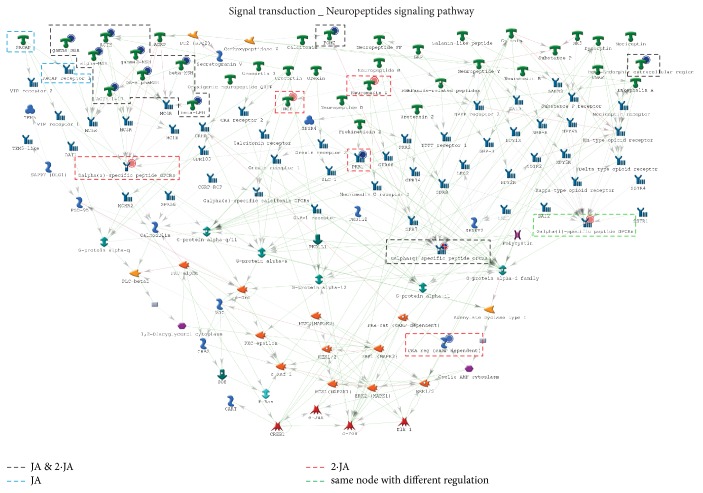
*Signal transduction-neuropeptides signaling pathways*. They contain 28 target genes, with 23 and 17 in JA and 2·JA, respectively. Among them, 12 target molecules were activated by both dosages (in black); 2 were only activated in the JA (in blue) group, while 5 were specific to 2·JA (in red) treatment.

Next, we focused on the* signal transduction-Neuropeptides signaling pathways*, which are the most enriched processes in both the 2·JA and JA groups. They contain 28 target genes, with 23 and 17 found in the JA and 2·JA groups, respectively. Among them, 12 target molecules were activated by both JA and 2·JA treatments (Figure 5, in black), including* gamma-MSH*,* alpha-MSH*, and* ACTH*;* PACAP* and* PACAP receptor 1* were only activated after JA treatment (Figure 5, in blue);* PKR1* and* PKA-re (cAMP-dependent)*, among others, were exclusively found in the 2·JA group (Figure 5, in red). Notably, 11 of the 12 target genes were downregulated in both groups. Further the* Galpha(i)-specific peptide GPCRs* was upregulated in the 2·JA group but downregulated in JA group, indicating that 2·JA could reverse target expression levels within the same process (Figure 5, in green). Besides, in the second significant process of* Development_Neurogenesis: Synaptogenesis* and the forth significant process of* cell adhesion_Amyloid proteins* for 2·JA group, several significant nodes were found, including* PKR 1*,* TBR1*, and* WNT*.

#### 2.4.3. The Molecular Function Distribution of JA and 2·JA Groups

The comparative analysis of the top 10 pathways and process networks of the 2 treatment groups found 3 common pathways and 4 common processes (Figures 2 and 3). Although common pathways and processes were identified, the differentially expressed genes in the 2·JA group were significantly different from those obtained after JA treatment.

Seven of the top 10 pathways in the 2·JA group were involved in neuroprotection, 40% in apoptosis and survival, 30% in the regulation of lipid metabolism, and 20% in anti-inflammatory reactions, all annotated by GeneGo. On the other hand, 40% of the top 10 pathways in the JA group were involved in neuroprotection and antiapoptosis, 10% in neuroprotection and anti-inflammation, and 10% in neuroprotection, antiapoptosis, and anti-inflammation.

### 2.5. Ischemia Pathways Observed in the Vehicle Group Were Not Activated in JA and 2·JA

**Figure 6:**
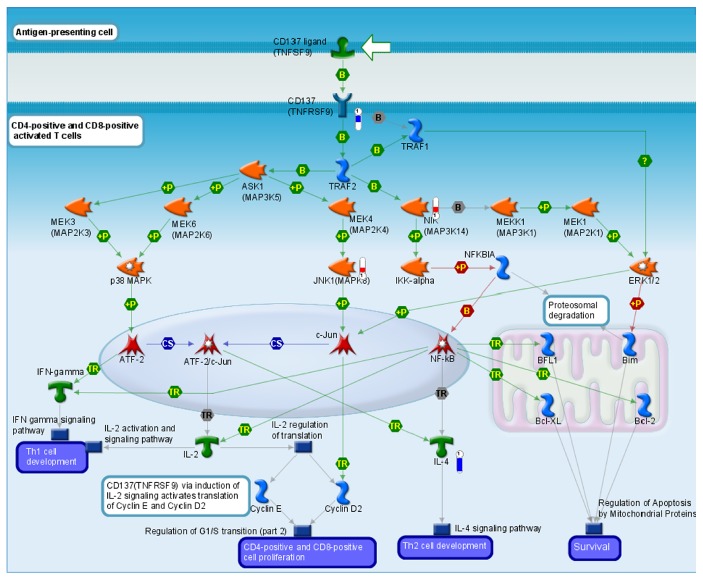
JNK1 and NIK pathways were upregulated and contributed to cell death in brain ischemia in the Vehicle group.

Seven of top 10 pathways in the Vehicle group were no longer activated by either JA or 2·JA. For example,* Immune response_CD137 signaling in immune cell* (Figure 6),* JNK1*, and* NIK* signaling pathways were activated in the Vehicle group. The activation of* JNK1* contributes to cell death in brain ischemia through both necrosis and apoptosis pathways [12]; and* NIK* blocks both classical and alternative nuclear factor-*κ*B* (NF-κB)* activation pathways and reduces the expression of several prosurvival and antiapoptotic factors in brain ischemia [13]. In* cell adhesion_Role of CDK5 in cell adhesion*, the upregulated activity of* CDK*5 was shown to contribute to neuronal death following ischemia [14]. These pathways were inhibited after treatment with either JA or 2·JA, demonstrating that pathways contributing to brain injury after ischemia in the Vehicle group were altered.

## 3. Discussion

In this study, gene expression microarrays and GeneGo systematic pathway analysis elucidated the dose-effect mechanism of 2·JA. Based on the enriched pathways, molecular processes, and key targets of JA and 2·JA, it was consistently shown that the molecular mechanisms underlying the 2·JA treatment of cerebral ischemia were significantly altered when compared to a single JA dose.

**Figure 7:**
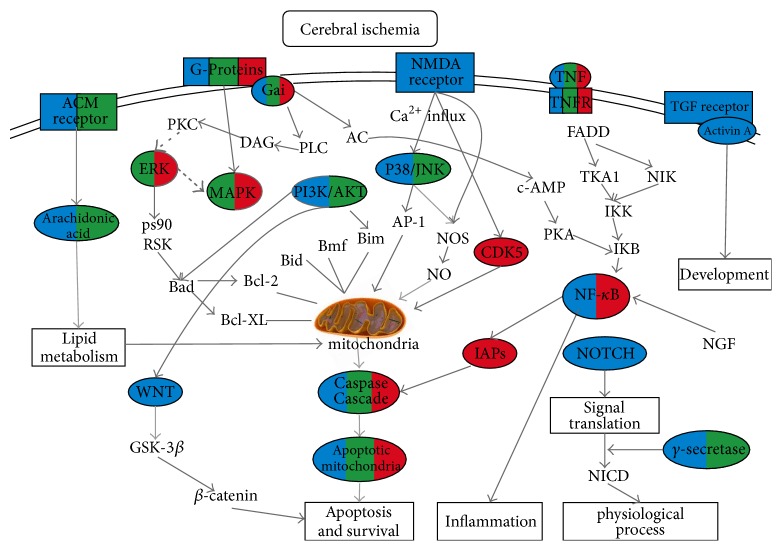
Altered genes representing 2·JA (in blue), JA (in green), and Vehicle (in red) that target pathways involved in cerebral ischemia. Lipid metabolism, apoptosis and survival, inflammation pathways, and others were affected.

Among the top 10 enriched pathways, 2·JA and JA both affected neuroprotection, anti-apoptosis, anti-inflammation and lipid metabolism. However, 2·JA treatment resulted in more pronounced effect on neuroprotection (70%) while JA was more anti-apoptotic (60%); 2·JA and JA played nearly the same role in anti-inflammation and regulation of lipid metabolism (Figure 7). These findings demonstrated that the altered dosage changes the function distribution, which may constitute a mechanism for double JA dosage's additive therapeutic effect in the treatment of cerebral ischemia.

Compared with JA, pathways specific to 2·JA were mainly involved in neuroprotection, apoptosis and survival, anti-inflammation, and regulation of metabolism. There is overwhelming evidence that these biological pathways are important therapeutic mechanism in ischemia therapy [15]. For example,* Gamma-Secretase regulation of neuronal cell development and function* is related to Gamma-Secretase, an important pathway that may help combat cerebral ischemia and that is discussed further ahead. Its regulation by 2·JA had antiapoptotic and neuroprotective effects on neuronal cells after ischemia by targeting apoptotic protease, cleaved* caspase-3*, and* NF-κB*, among others [16].* Development_Role of Activin A in cell differentiation and proliferation *was another 2·JA's signature pathway. Activin A, a member of the transforming growth factor-beta (TGF-b) family, is an endogenous neuronal survival factor increased by 2·JA; after acute brain injury, it may have proven beneficial therapeutic activity against cerebral ischemia [17]. Indeed, Activin A protects neurons against oxidative challenge and promotes tissue survival after focal cerebral ischemia/reperfusion, with an extended therapeutic window [18]. In* Cytoskeleton remodeling_TGF-β, WNT*,* and cytoskeletal remodeling*, 2·JA regulated the* TGF-β *and* WNT *signaling pathways. Studies have indicated that increased* TGF-β* signaling after stroke is neuroprotective and likely to be an important target for future stroke therapies [19, 20].* WNT* signaling can enhance neurogenesis and improve neurological function after ischemic injury [21, 22]; it also has anti-inflammatory effects [23] and is neuroprotective in stroke [24]. Besides,* Atherosclerosis_Role of ZNF202, Regulation of metabolism_Bile acids, regulation of glucose, and* lipid metabolism via FXR, Neurophysiological process_HTR1A receptor signaling in neuronal cells, are also pathways that play important roles in neuroprotection [25–28]. Taken together, the pathways exclusively identified in the 2·JA group were mostly related to inflammatory response, neuroprotection, apoptosis, and survival, which may constitute the double dosage additive mechanism of JA in the treatment of cerebral ischemia.

As mentioned above, the significant nodes* ADAM10, APP, Amyloid beta, AICD, CTFs*, and* NMDA receptor* may be the key targets related to 2·JA effect (Figure 4), since their expression level changing activated 2 of the top 10 pathways, including* Gamma-Secretase proteolytic targets* and* Gamma-Secretase regulation of neuronal cell development and function*.* ADAM10* has been shown to mediate repair in response to neuronal damage in the brain [29]; it is an important enzyme for proteolytic processing of amyloid precursor protein* (APP)* [30]. Cleavage products of* APP* include* Amyloid beta*,* alpha APPs*,* beta APPs*,* AICD*, and* CTFs*. Several studies have demonstrated the neuroinflammatory effects of* Amyloid beta* [31];* alpha APPs* have been reported to have neurotrophic and neuroprotective properties;* beta APPs* seemed to have a proapoptotic function [31].* AICD* (amyloid precursor protein intracellular cytoplasmic/C-terminal domain) regulates* Amyloid beta* degradation [32]; meanwhile, degradation of* CTFs* (APP C-terminal fragments) can suppress* Amyloid beta* generation [32, 33]. The common variants of* Amyloid beta *are* amyloids beta 40 and 42*, of which only the monomeric forms exhibit neuroprotective and antioxidant effects [34]. It has also been shown that* NMDA receptor* activation leads to* ADAM10* upregulation and induces* APP* breakdown [30]. These important nodes may be the potential targets of 2·JA, providing neuroprotection and antiapoptotic effects in the treatment of cerebral ischemia.

With reference to the process networks, both 2·JA and JA acted on* signal transduction-neuropeptides signaling pathways* and the differential expressed genes, such as* alpha-MSH, ACTH, GPCRs, beta-LPH, *and* POMC*, which play important roles in ischemia injury. The family of* GPCRs* involved in the* c-AMP/PKA* pathway is known to provide neuroprotection against ischemic injury [35] and activate* CREB* for neuronal cell-survival after ischemia injury [36].* Alpha-MSH* confers anti-inflammatory [37], antioxidative, and antiapoptotic [38] effects after ischemia. In addition, the neuroprotective, anti-inflammatory, and antiapoptotic effects of* ACTH *have been demonstrated [39, 40]. In the JA group,* PACAP*, an exclusive target, is involved in antiapoptosis [41] and neuroprotection [42]; its receptor* PACAP receptor 1* is neuroprotective [43].* Neuromedin U, PKR 1, PKA-reg (cAMP-dependent), APOE, *and* TBR1 *may constitute key nodes related to 2·JA's effect.* PKR1* is involved in angiogenesis and inflammation [44, 45]. The neuron-specific transcription factor* TBR1 (T-box brain 1)* is involved in the second process* Development_Neurogenesis: Synaptogenesis *and is known to regulate brain development [46] and to play a role in adaptive cytoskeletal remodeling [47]. Targets with altered expression in* cell adhesion_Amyloid proteins*, the forth process, were also noteworthy:* Amyloid beta, ADAM10, APP, alpha APPs*, and* WNT *are related to neuroprotection, anti-inflammation, and antiapoptosis. These important nodes are potential targets involved in the pharmacological mechanism of 2·JA in the treatment of cerebral ischemia.

In future investigations, the differential expression of some of these target genes should be tested quantitatively. Ideally, the expression of main molecules (ligands, receptors, and effectors such as transcription factors) of the more relevant pathways and networks associated with each therapeutic group (JA and 2·JA) will be quantitatively monitored. Such data would enable further understanding of the pharmacological mechanism and potential targets of JA's dosage effect.

## 4. Conclusion

System-based pathway and network analysis of transcriptomic microarrays revealed that 7 pathways and 3 process networks of the 10 most significantly regulated after 2·JA treatment mainly affect neuroprotection (70%), apoptosis and survival (40%), and anti-inflammation (20%). These pathways, which include Gamma-Secretase regulation of neuronal cell development and function, alpha-MSH, ACTH, PKR1, and WNT, and the overall altered functional profiles reveal the molecular mechanism of 2·JA dosage's effect in treatment of cerebral ischemia.

## 5. Methods

### 5.1. Animals

A total of 48 healthy specific pathogen-free adult male Kunming mice (12 weeks old, 38 to 48 g) were housed at 25°C in a 12-hour light/dark cycle environment. The exploratory assessment of dose-effect relationship focused on individual- and double-JA dosages. Therefore, mice were randomly divided into 4 groups (each consisting of 12 samples): Sham, Vehicle, JA, and 2·JA. Protocols for animal use were reviewed and approved by the Ethics Review Committee for Animal Experimentation of the China Academy of Chinese Medical Sciences; all animal experiments were conducted in accordance with the Prevention of Cruelty to Animals Act of 1986 and the National Institute of Health guidelines for care and use of experimental laboratory animals.

### 5.2. Middle Cerebral Artery Occlusion

For the Vehicle, JA, and 2·JA groups, mice were intraperitoneally anaesthetized with 2% pentobarbital (40 mg/Kg) and surgery was carried out to induce middle cerebral artery occlusion (MCAO). The focal cerebral ischemia-reperfusion model was induced by occluding the left middle cerebral artery for 1.5 h with an intraluminal filament followed by reperfusion for 24 h. For sham treated animals, the external carotid artery was surgically prepared, but no filament was inserted. During the experimental procedures, blood pressure, blood gas levels, and glucose amounts were monitored; in each animal, rectal temperature was maintained at 37.0–37.5°C with a heating pad; body temperature was kept at 37°C with a thermostatically controlled infrared lamp; brain temperature (monitored with a 29-gauge thermocouple in the right corpus striatum) was maintained at 36-37°C with a temperature-regulating lamp. An electroencephalogram was acquired to ensure isoelectricity during the ischemic period. Operational success was determined based on infarct volume. The infarct volume of each group was calculated using a Pathology Image Analysis System (Topica Inc.), and the ratio of the infarct volume to the total slice was calculated.

### 5.3. Drug Administration

The animals were treated as follows: sham group: 0.9% NaCl; Vehicle group: 0.9% NaCl; JA group: 25 mg/ml Jasminoidin; 2·JA group: 50 mg/ml Jasminoidin. The drug preparation used was a chemically standardized product obtained from the National Institutes for Food and Drug Control, which was validated by fingerprint chromatographic methodologies. The herbal preparations were dissolved in 0.9% NaCl immediately before use. All preparations were administered by tail vein injection at the same time point, 1.5 h after focal cerebral ischemia induction at 2 ml/kg. The preparations were chemically standardized products obtained from the China Natural Institute for the Control of Pharmaceutical and Biological Products or the Beijing University of Traditional Chinese Medicine.

### 5.4. RNA Isolation

The left hippocampus samples of 9 mice per group were homogenized in TRIzol® Reagent (Invitrogen, USA). According to the manufacturer's instructions, total RNA was extracted using the RNeasy Micro Kit (Qiagen, Valencia, CA). The RNA integrity was determined by the 26S/18S ratio, using a Bioanalyzer microchip device (Agilent, Palo Alto, CA, USA).

### 5.5. Microarrays

Gene expression profile was assessed using a mouse brain array (BoaoCapital, Beijing, China), which consists of 374 stroke-related cDNAs, in-house prepared with a microarray chip for the whole mouse genome (Incyte Genomics, Santa Clara, CA, USA). Duplicate clones were imprinted on each chip, generating 4 technical replicates per clone. A single intensity value for each clone was generated by averaging quadruplet measurements after smoothing spline normalization. All clones were verified by DNA sequencing. RNA from the Vehicle group was pooled and labelled with Cy3, while Cy5 was used for the other groups. Microarrays were hybridized, washed, and scanned according to standard protocols. For each group, the above procedures were repeated in at least biological triplicates and technical quadruplets.

### 5.6. Microarray Data Analysis

All experimental data were uploaded into the ArrayTrack system. Experimental analysis was based on the Minimum Information about Microarray Experiment Guidelines and the Microarray Quality Control Project, and the results were submitted to the Array Express database (Accession number: E-TABM-612). All microarray data were normalized by locally weighted linear regression to reduce experimental variability (smoothing factor: 0.2; robustness iterations: 3). One-way analysis of variance (ANOVA) and Significance Analysis of Microarrays (SAM) were used to compare the means of genes altered between JA or 2·JA treated animals and the Vehicle group. The differentially expressed genes (DEGs, *p* < 0.05) were selected for further analyzed; the DEGs of JA and 2·JA were listed in Supplementary Table 1. This cutoff value was set to identify molecules significantly differentially regulated, which were labelled as Network Eligible Molecules (NEMs). Networks of NEMs were algorithmically generated based on connectivity. Right-tailed Fisher's exact test was used to calculate the probability (*p* value) that each biological function assigned to a network was due to chance alone. The significance of the association between these genes and the canonical pathways was measured by one of the following 2 ways:

(1) A ratio of the number of genes from the dataset mapping the pathway by the total number of genes mapping to the canonical pathway

(2) A *p* value, calculated by Fisher's exact test, determining the probability that the association between the genes and canonical pathway was explained by chance alone. The level of statistical significance was set at 0.05. Finally, canonical pathways with *p* < 0.05 and a log fold change > 1.5 screened and analyzed.

### 5.7. Network Calculation of Enrichment

Enrichment analysis is a computational method for identifying the functional distribution of genomic/proteomic expression profiles and significantly enriched functional categories [48]. Biological processes, subcellular locations, and molecular function distributions of differentially expressed genes were computed using MetaCore based on Gene Ontology annotations [49]; the network distribution of selected genes was computed using MetaCore based on GeneGo network ontologies.

### 5.8. Statistical Analysis

Significance of enrichment and pathways was evaluated with scores produced using the Expression Analysis Systematic Explorer software (National Institute of Health, USA) [50], which employs modified Fisher's exact test [51]. To assess pathways, the statistical significance of ontology matches was evaluated as the probability of a match to occur by chance, taking database size into account. Lower significance, which denoted higher ratings for matched terms, was expected as the number of genes/proteins belonging to a single process/pathway increased, with *p* < 0.05 considered statistically significant.

## Data Availability

The microarray data used to support the findings of this study have been deposited in the ArrayExpress repository (E-TABM-612).

## Supplementary Materials

Supplementary Materials Supplementary Table 1: there are differentially expressed genes (DEGs) in JA and 2·JA groups.
